# Airway epithelial cell exposure to distinct e-cigarette liquid flavorings reveals toxicity thresholds and activation of CFTR by the chocolate flavoring 2,5-dimethypyrazine

**DOI:** 10.1186/s12931-016-0369-9

**Published:** 2016-05-17

**Authors:** Cara L. Sherwood, Scott Boitano

**Affiliations:** Asthma and Airway Disease Research Center, Arizona Health Sciences Center, 1501 N. Campbell Avenue, Tucson, AZ 85724-5030 USA; Bio5 Collaborative Research Institute, Arizona Health Sciences Center, Tucson, AZ USA; Department of Physiology, Arizona Health Sciences Center, Tucson, AZ USA

**Keywords:** Electronic cigarettes, Airway epithelium, CFTR, Odorant receptor, 2,5-dimethylpyrazine, xCELLigence RTCA

## Abstract

**Background:**

The potential for adverse respiratory effects following exposure to electronic (e-) cigarette liquid (e-liquid) flavorings remains largely unexplored. Given the multitude of flavor permutations on the market, identification of those flavor constituents that negatively impact the respiratory tract is a daunting task. In this study we examined the impact of common e-liquid flavoring chemicals on the airway epithelium, the cellular monolayer that provides the first line of defense against inhaled particulates, pathogens, and toxicants.

**Methods:**

We used the xCELLigence real-time cell analyzer (RTCA) as a primary high-capacity screening tool to assess cytotoxicity thresholds and physiological effects of common e-liquid flavoring chemicals on immortalized human bronchial epithelial cells (16HBE14o-). The RTCA was used secondarily to assess the capability of 16HBE14o- cells to respond to cellular signaling agonists following a 24 h exposure to select flavoring chemicals. Finally, we conducted biophysical measurements of well-differentiated primary mouse tracheal epithelial (MTE) cells with an Ussing chamber to measure the effects of e-cigarette flavoring constituents on barrier function and ion conductance.

**Results:**

In our high-capacity screens five of the seven flavoring chemicals displayed changes in cellular impedance consistent with cell death at concentrations found in e-liquid. Vanillin and the chocolate flavoring 2,5-dimethylpyrazine caused alterations in cellular physiology indicative of a cellular signaling event. At subcytotoxic levels, 24 h exposure to 2,5-dimethylpyrazine compromised the ability of airway epithelial cells to respond to signaling agonists important in salt and water balance at the airway surface. Biophysical measurements of 2,5-dimethylpyrazine on primary MTE cells revealed alterations in ion conductance consistent with an efflux at the apical airway surface that was accompanied by a transient loss in transepithelial resistance. Mechanistic studies confirmed that the increases in ion conductance evoked by 2,5-dimethylpyrazine were largely attributed to a protein kinase A-dependent (PKA) activation of the cystic fibrosis transmembrane conductance regulator (CFTR) ion channel.

**Conclusions:**

Data from our high-capacity screening assays demonstrates that individual e-cigarette liquid flavoring chemicals vary in their cytotoxicity profiles and that some constituents evoke a cellular physiological response on their own independent of cell death. The activation of CFTR by 2,5-dimethylpyrazine may have detrimental consequences for airway surface liquid homeostasis in individuals that use e-cigarettes habitually.

## Background

Electronic (e-) cigarettes are the most common type of electronic nicotine delivery systems (ENDS) that simulate smoking independent of the combustion of tobacco. The global market for ENDS has rapidly expanded and it is predicted that within the next decade, sales of ENDS will surpass that of conventional combustible tobacco cigarettes [1]. In the U.S. market alone e-cigarette sales are estimated to be $10 billion by 2017 [2]. As of 2014 there were >450 distinct e-cigarette products with >7500 flavor variations available for sale online and/or at retail outlets worldwide [3–6]. Despite the rapid increase in popularity of ENDS the potential for harmful respiratory effects following use of these products remains largely unexplored.

In their simplest form e-cigarettes contain a fluid-filled (e-liquid) cartridge with a battery-powered atomizer. E-liquid comes in a vast variety of configurations and can contain numerous ingredients including vegetable glycerin (VG) responsible for the visible vapor, the humectant propylene glycol (PG), nicotine, menthol and/or other flavorings. Although an early study of e-cigarettes suggested that the levels of several harmful constituents are lower in e-cigarette aerosols when compared with cigarette smoke [7], some constituents unique to e-cigarette aerosols, namely flavorings, have been shown to be cytotoxic in cell models [8].

Research in to the composition and concentration of flavorings in e-liquids is just beginning [9–11]. E-liquid flavorings are often advertised as “safe” because they are approved for ingestion. However, the airway and the gastrointestinal tract are quite distinct and represent significantly different toxicity susceptibilities. A cautionary example that illustrates this point is the toxicity fate of diacetyl, an early component of artificial butter popcorn flavoring. Despite being approved for ingestion, excessive inhalation exposure of diacetyl to lung tissue results in bronchiolitis obliterans, a rare and irreversible lung disease [12]. The potential for e-liquid flavoring chemicals to cause detrimental health effects warrants their investigation [13].

The conducting airway epithelium provides the first line of defense against inhaled particulates, pathogens, allergens, and other noxious agents [14]. The varied epithelial cell types along the conducting airway provide key innate immune functions including: a physical barrier to protect the underlying tissue; salt and water movement to maintain a hydrated lumen; a mucociliary escalator to coordinate particulate filtering; and secretion of multiple defense factors such as antimicrobials. These functions of airway epithelial innate immunity are facilitated by signal transduction molecules such as ATP and cAMP. A compromised airway epithelium can lead to infection, inflammation and airway remodeling associated with the onset and pathogenesis of chronic lung disease (reviewed in [15–19]). Because e-cigarette aerosols are delivered directly to the airway a logical place to initiate toxicity studies is with airway epithelial cell models.

In this study we used high-capacity real-time cell analysis as a primary screen to identify toxicity thresholds of e-liquid flavorings on human bronchial epithelial cells (16HBE14o-). Because cells can contribute to disease in lieu of cell death, we also used high-capacity real-time cell analysis to measure responses to cellular signaling molecules (i.e., ATP and forskolin-induced cAMP) following exposure to subcytotoxic levels of e-liquid flavoring chemicals. From the cytotoxic and subcytotoxic profiles established, we selected 2,5-dimethylpyrazine for more thorough mechanistic studies. From biophysical analyses we showed a direct effect of 2,5-dimethylpyrazine on the regulation of Cl^-^ secretion. These findings confirm the need for high-capacity toxicity screening that can lead to mechanistic understanding to better predict risks associated with the rapidly growing e-cigarette products.

## Methods

### Materials

Cellgro DMEM:F12 was from Mediatech (Manassas, VA). Lechner and LaVeck basal media (LHC), Hanks’ Balanced Saline Solution, glutamax, penicillin, and streptomycin were from Life Technologies (Carlsbad, CA). Fibronectin, type I collagen, and Nu-Serum™ were from Becton-Dickinson (Franklin Lakes, NJ). Minimum Essential Medium with Earle’s salts (MEM), Fetal Bovine Serum (FBS), 2,5-dimethylpyrazine, amiloride, damascenone, forskolin, linalool, α-ionone, ethyl maltol, furaneol and vanillin were from Sigma-Aldrich (St. Louis, MO). CFTR-172 inh and 8-bromo-cAMP were from Tocris Bioscience (Bristol, UK). Semipermeable filters were Corning Costar 6.5 mm Transwell® with 0.4 μm Pore Polyester Membrane Insert, sterile (Lowell, MA). All other chemicals were from Sigma-Aldrich or Fisher Scientific (Pittsburgh, PA).

### Immortalized human bronchial epithelial cell culture methods

16HBE14o- cells, a SV40 transformed human bronchial epithelial cell line [20], were obtained through the California Pacific Medical Center Research Institute (San Francisco, CA, USA). Growth conditions for 16HBE14o- cells have been described [21, 22]. 16HBE14o- cells were grown on a collagen/fibronectin/BSA (CFB) matrix. Cells were expanded in flasks and passaged onto E-plates (ACEA Biosciences, San Diego, CA) at 40,000 cells per well for high-capacity real-time cell analysis.

### Primary Mouse Tracheal Epithelial (MTE) cell culture methods

Animal protocols were approved by the Institutional Animal Care and Use Committee of The University of Arizona. Primary MTE cells were chosen for Ussing chamber studies because they are representative of a polarized epithelium necessary for biophysical measurements. C57Bl/6 wild type mice were used for these cells that were cultured as previously described [23]. MTE cells were seeded onto 6.5 mm semipermeable filters coated with CFB matrix and cultured at 37 °C with 5 % CO_2_. After cell monolayers reached a transepithelial resistance of > 500 Ω ⋅ cm^2^ (~5 days) the apical media was removed to establish an air/liquid interface (ALI). These cells become a mixed population of well-differentiated cells with clearly established cilia after ~ 5 days at ALI. Cells were used for experimentation 2–3 weeks after differentiation was established.

### xCELLigence real time cell analyzer toxicity and cell signaling assays

Methods for high-capacity real-time cell analysis of toxicity and physiological responses have been described [21, 24] and the measurement of cytotoxicity validated in 16HBE14o- cells [25]. 16HBE14o- cells were plated in full culture medium onto 96 well E-plates coated with CFB solution and allowed to grow at 37 °C and 5 % CO_2_ while impedance at the well surface was continuously monitored [26, 27]. As per manufacturer’s instructions (ACEA Biosciences, San Diego, CA), relative impedance is expressed as a Cell Index where: Cell Index = (Z_i_-Z_0_)/15Ω; and Z_i_ is impedance at a given time point during the experiment (i.e., post ATP addition), and Z_0_ is impedance before the addition of agonist. For reference, a dramatic decrease in impedance can be indicative of cell death whereas activation of GPCR/G_q_, such as occurs following ATP activation of purinergic receptors, results in an increase in Cell Index [21]. To determine toxicity thresholds cells were grown overnight on an E-plate and then exposed to varying doses of e-liquid flavoring chemicals diluted in full culture medium. Cell Index responses to e-liquid flavorings were recorded every 15 min for 24 h. Physiological responses to ATP and forskolin were recorded every 30 s for 4 h following a 24 h exposure to select e-liquid flavorings. Cell responses were collected in triplicate or quadruplicate. To better compare readings, cell responses were adjusted to a baseline by taking the ratio of recordings from cells in culture medium alone and then normalizing at the time point of e-liquid flavoring or cell signaling agonist addition as described in supplemental figure S2 in [28]. E-liquid flavoring chemicals screened with high-capacity real-time cell analysis were based on those from popular brands such as blu and GreenSmoke® e-cigarette cartridges with distinct organoleptic properties including: 2,5-dimethylpyarzine (chocolate, nutty flavor); damascenone (apple, citrus, wine-like); linalool (floral, spice); α-ionone (fruity, raspberry); ethyl maltol (caramel); furaneol (strawberry, sweet); and vanillin (vanilla) [29, 30].

### MTS cytotoxicity assay

16HBE14o- cells were seeded on a tissue culture treated 96-well plate coated with CFB at a density of 40,000 cells/well. Cells were grown overnight and then treated with e-liquid flavoring chemicals diluted in MEM. Following a 24-h exposure to the e-liquid chemicals cytotoxicity was assessed using a CellTiter 96® AQueous Non-Radioactive Cell Proliferation Assay (MTS; Promega, Madison, WI). MTS dye was added to all wells at a 1:10 dilution and allowed to incubate at 37 °C in 5 % CO_2_ for 1.5 h. The 96-well plate was then shaken vigorously and read in a Synergy™ HTX Multi-Mode Microplate Reader (BioTek, Winooski, VT) at an absorbance of 490 nm.

### Ussing chamber studies

MTE cells on 6.5 mm permeable filters were mounted in an EasyMount Ussing chamber system (Physiologic Instruments, San Diego, CA) and bathed on both sides with Krebs-Ringers Buffer containing: 115 mM NaCl, 25 mM NaHCO_3_, 0.4 mM KH_2_PO_4_, 2.4 mM K_2_HPO_4_, 1.2 mM CaCl_2_, and 1.2 mM MgCl_2_ with 10 mM glucose. Bath solutions were continuously circulated with a gas lift by bubbling with 95 % air and 5 % CO_2_ at 37 °C (pH 7.4). MTE cell monolayers were voltage clamped and monitored for changes in short-circuit current (I_sc_) and TER with a multichannel voltage/current clamp VCC MC8 (Physiologic Instruments, San Diego, CA). MTE cell monolayers were clamped to 0 mV and a 5 mV pulse of 200-msec duration was imposed every 10 s. Changes in I_sc_ (ΔI_sc_) were calculated from the difference between the initial I_sc_ measurement at baseline and the peak measurement change after adding 2,5-dimethylpyrazine alone or in combination with pharmacological inhibitors/agonists. Data was analyzed using Acquire and Analyze software, version 2.3 (Physiologic Instruments, San Diego, CA).

### Statistics

One-way ANOVA with a Dunnett’s Multiple Comparison Test was used for statistical analysis unless otherwise noted. A value of *P* < 0.05 was used to establish significant differences between data sets. Figures are graphed ± standard error of the mean (SEM) unless otherwise noted.

## Results

### High-capacity real-time cell analysis reveals toxicity thresholds of e-liquid flavorings

**Fig. 1:**
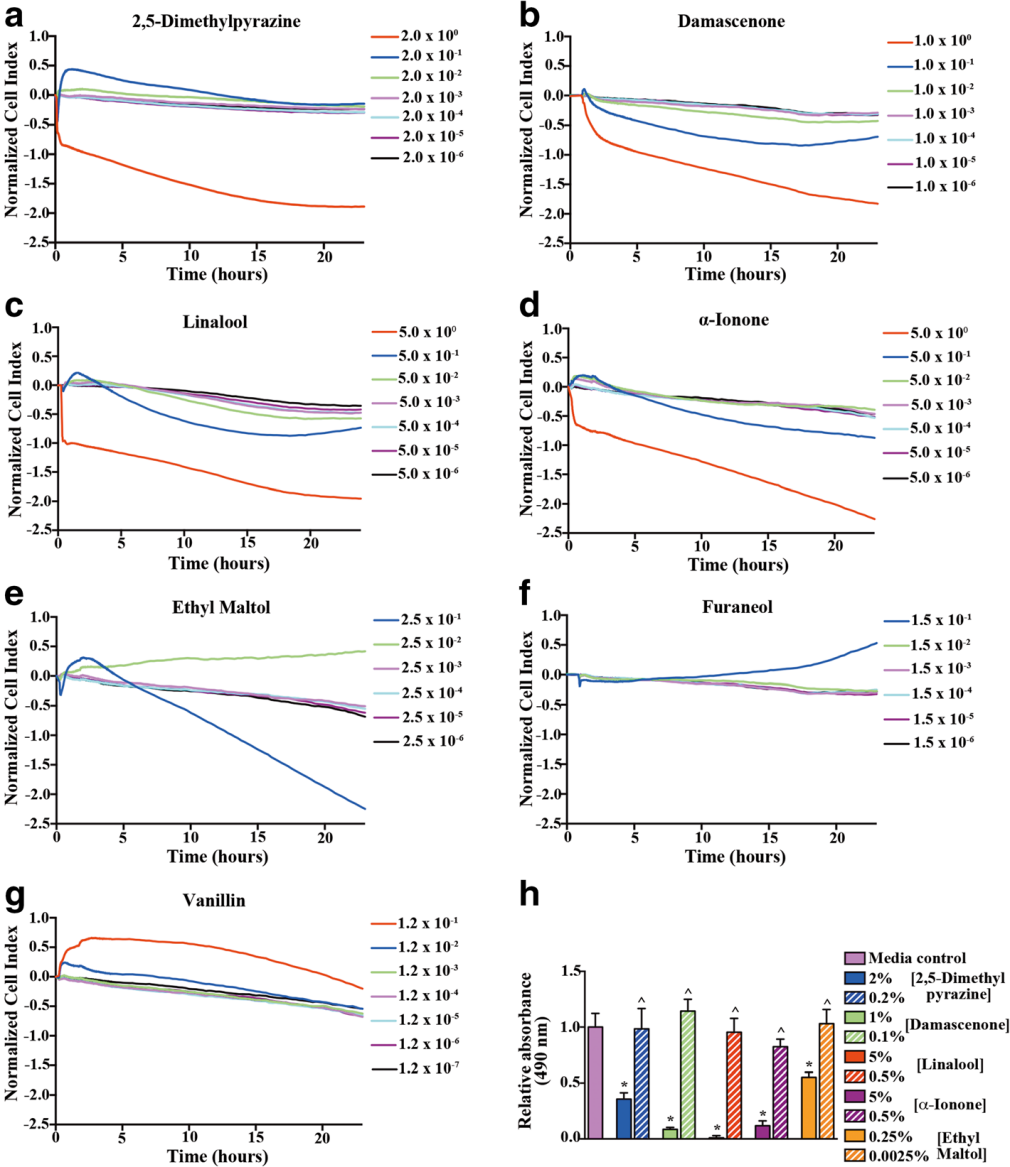
High-capacity real-time cellular analysis reveals toxicity thresholds of e-liquid flavorings. The toxicity thresholds of individual e-cigarette liquid flavoring constituents on 16HBE14o- cells was determined by cellular impedance changes over 24 h. **a**-**d** The highest concentration of each flavoring constituent was representative of the % v/v present in e-cigarette liquid. **e**-**g** The highest concentration of each flavoring constituent represented 1/10th the % v/v present in e-cigarette liquid. All flavors except furaneol (**f**) and vanillin (**g**) displayed cell index changes consistent with cytotoxicity that was validated with an MTS assay (**h**). *indicates significant difference from control cells and ^indicates significant difference between concentrations of the same flavoring, *P* < 0.05 in a one-way ANOVA with a Tukey’s Multiple Comparison Test

Elucidating e-liquid flavorings that pose potential risks to lung health is challenging given the vast array of available flavoring chemical constituents. To address this barrier we used high-capacity real-time cell analysis on a human bronchial epithelial cell line (16HBE14o) with multiple e-liquid flavoring chemicals. We screened seven flavoring chemicals present in e-liquid cartridges from popular e-cigarette brands (i.e., Blu, GreenSmoke®) [29, 30]. Four of the flavorings (Fig. 1a-d) were tested with the highest concentration representing the percent volume/volume (% v/v) present in GreenSmoke® e-cigarette cartridges and three flavorings (Fig. 1e-g) were tested starting at a concentration one-tenth (due to solubility issues) the % v/v present in GreenSmoke® e-cigarette cartridges. All flavorings were screened at multiple concentrations in quadruplicate.

In five of the seven compounds tested the highest concentration(s) displayed a loss of cell index consistent with cytotoxicity (Fig. 1a-e). We confirmed the loss of cell index was due to cytotoxicity with an MTS assay. In the MTS assay we compared those e-liquid chemical concentrations that showed cytotoxicity in the RTCA with the same flavor chemicals at a concentration that did not display cytotoxicity in the RTCA (Fig. 1h). Furaneol (Fig. 1f) and vanillin (Fig. 1g) did not display any cell index changes consistent with cell death; however, the cell index for the full concentration present in e-liquid for these compounds could not be tested due to solubility issues. In addition to cytotoxicity responses at the higher concentrations, at dilutions to one-tenth the amount present in e-liquid, 2,5-dimethylpyrazine and vanillin displayed increases in cell index consistent with a cell-signaling event. Because of the unique combination of cytotoxicity and cellular signaling exhibited by 2,5-dimethylpyrazine we selected this compound from the initial screen for further toxicity studies.

### 2,5-Dimethylpyrazine reduces the physiological response to signaling molecules important in aspects of airway epithelial cell innate immunity

**Fig. 2:**
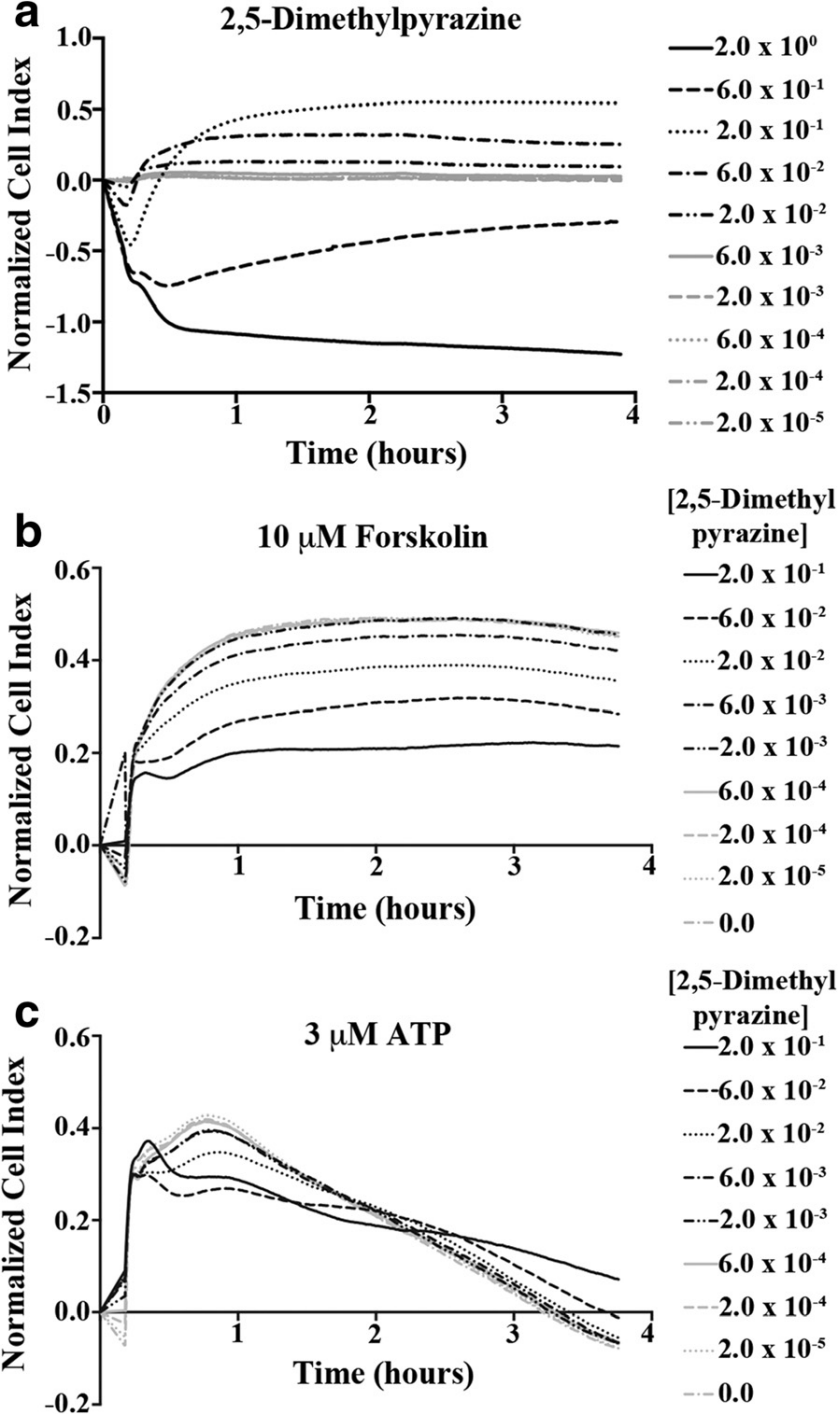
2,5-Dimethylpyrazine reduces the physiological response to cellular signaling critical in airway innate immunity. **a** 16HBE14o- cells display cytotoxic cellular impedance responses to 2,5-dimethylpyrazine at 0.6 % and higher. **b** A 24 h exposure to subcytotoxic concentrations of 2,5-dimethylpyrazine causes a concentration-dependent signaling response to forskolin in 16HBE14o- cells. **c** A 24 h exposure to subcytotoxic concentrations of 2,5-dimethylpyrazine results in a reduction in 16HBE14o- signaling response to ATP. At subcytotoxic concentrations, 2,5-dimethylpyrazine evoked a physiological response at concentrations as low as 0.06 % v/v and dampened the response to cellular signaling key in airway epithelial innate immunity

We conducted a secondary high-capacity real-time cell analysis screen of 2,5-dimethylpyrazine to examine in finer detail the signaling pattern elicited by 16HBE14o- cells upon exposure (Fig. 2a). Concentrations of 2,5-dimethylpyrazine as low as 0.02 % v/v (1/100 of what is in e-liquid) induced distinct cellular impedance changes indicative of a cellular signaling event. Additionally, we tested the capability of 16HBE14o- cells to respond to cAMP (via forskolin) or exogenous ATP, important signaling molecules in airway epithelial cell physiology (e.g., mucociliary clearance), following exposure to 2,5-dimethylpyrazine (Fig. 2b-c). 16HBE14o- cells were treated for 24 h with subcytotoxic concentrations of 2,5-dimethylpyrazine followed by exposure to either forskolin (10 μM; to raise cAMP) or exogenous ATP (3 μM; to raise intracellular Ca^2+^ concentration) and cellular impedance monitored. Compared to untreated cells, cells that were treated for 24 h with 2,5-dimethylpyrazine displayed a concentration-dependent reduction in physiological response to both forskolin (Fig. 2b) and to ATP (Fig. 2c). We concluded that 2,5-dimethylpyrazine alters the capability of airway epithelial cells to respond to signaling molecules key in the proper functioning of airway cell physiology.

### 2,5-Dimethylpyrazine increases airway epithelial ion conductance

**Fig. 3:**
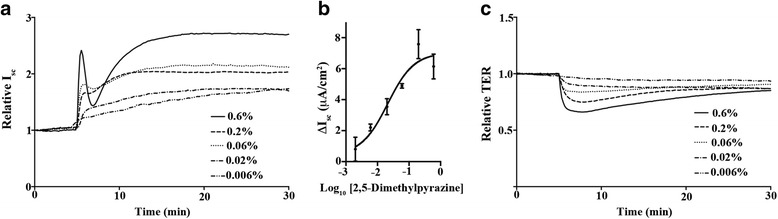
2,5-Dimethylpyrazine increases airway epithelial ion conductance. Primary MTE cells were voltage clamped in Ussing chambers and monitored for changes in I_sc_ and TER following varying doses of 2,5-dimethylpyrazine. **a** Representative traces of I_sc_ in response to 2,5-dimethylpyrazine demonstrate a concentration dependent increase in I_sc_. **b** A concentration-dependent curve of the change in I_sc_ following 2,5-dimethylpyrazine. **c** Representative traces of TER in response to varying concentrations of 2,5-dimethylpyrazine demonstrate a concentration dependent drop in TER. Addition of 2,5-dimethylpyrazine on the apical airway epithelial surface results in an increase in apical ion efflux with a concurrent drop in TER

In order to determine the impact of 2,5-dimethylpyrazine on airway epithelial ion conductance and barrier function we conducted Ussing chamber studies. Well-differentiated primary mouse tracheal epithelial (MTE) cells that retain characteristics such as polarization needed for biophysical measurements were mounted in Ussing chambers and voltage clamped for measurement of ion conductance [short circuit current (I_sc_)] and transepithelial resistance (TER). When administered in the apical chamber (i.e., luminal side of the epithelium) 2,5-dimethylpyrazine induced an apical ion efflux (Fig. 3a) that was accompanied by a transient loss in TER (Fig. 3c). Application of multiple concentrations of 2,5-dimethylpyrazine resulted in an I_sc_ EC_50_ ~ 1/100th of the concentration found in GreenSmoke® e-liquid (Fig. 3b; EC_50_ = 0.021 % v/v 95 % Confidence Interval: 0.0085 to 0.053 % v/v).

### 2,5-Dimethylpyrazine activates apical ion efflux via cystic fibrosis transmembrane conductance regulator (CFTR)

It is known that 2,5-dimethylpyrazine is an odorant compound found in dark chocolate [31]. Odorants can bind a class of G protein-coupled receptors that initiate an adenylyl cyclase-signaling cascade [32, 33]. Since airway epithelial cells express CFTR on their apical surface we tested the hypothesis that observed 2,5-dimethylpyrazine increases in I_sc_ (Fig. 3) were due to CFTR-mediated Cl^-^ conductance resulting from increased levels of cAMP downstream of odorant receptor activation. We conducted Ussing chamber studies under voltage clamp conditions with 0.2 % v/v 2,5-dimethylpyrazine, a subcytotoxic concentration that showed signaling activity in high-capacity real-time cell analysis and Ussing chamber studies, to evaluate the effects of pharmacological blockers/agonists in the cAMP/CFTR signaling cascade.

**Fig. 4:**
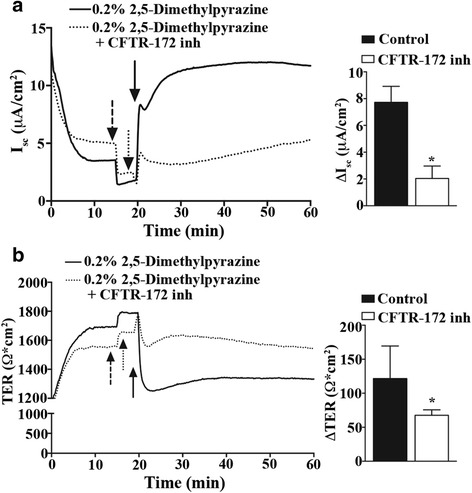
2,5-Dimethylpyrazine activates apical ion efflux via CFTR. Primary MTE cells were voltage clamped in Ussing chambers and monitored for changes in I_sc_ and TER following addition of 0.2 % v/v 2,5-dimethylpyrazine. **a** Representative traces of I_sc_ and **b** TER (*left*) from MTE cells treated with 2,5-dimethylpyrazine alone (solid line) or 2,5-dimethylpyrazine plus CFTR-172 inh [amiloride (dashed arrow), CFTR-172 inh (dotted arrow), 2,5-dimethylpyrazine (solid arrow)]. Quantification of I_sc_ and TER (A-B right) of amiloride controls (*n* = 3) versus cells treated with amiloride and CFTR-172 inh (*n* = 4) are graphed. The addition of CFTR-172 inh significantly reduced the 2,5-dimethypyrazine-evoked apical ion efflux suggesting Cl^−^ secretion via CFTR. *indicates significant difference *P* < 0.05 in a one-way ANOVA with a Dunnett’s Multiple Comparison Test

In the first experiment, we pre-treated MTE cell cultures with the epithelial sodium channel blocker amiloride (10 μM; Fig. 4a-b dashed arrow) followed by apical addition of CFTR blocker CFTR-172 inh (20 μM; Fig. 4a-b dotted arrow). These cells were then exposed to apical addition of 2,5-dimethylpyrazine (0.2 % v/v; Fig. 4a-b solid arrow) while monitoring I_sc_ and TER. The peak change in I_sc_ and TER evoked by 2,5-dimethylpyrazine in MTE cells pre-treated with amiloride and CFTR-172 inh (Fig. 4a-b dotted traces) was significantly reduced [Fig. 4a-b; ΔI_sc_: 2.0 ± 0.46 μA/cm^2^ (*n* = 4); ΔTER: 67.6 ± 4.0 Ω ⋅ cm^2^ (*n* = 4)] compared to those treated with only amiloride and 2,5-dimethylpyrazine [Fig. 4a-b solid traces; ΔI_sc_: 7.7 ± 0.69 μA/cm^2^ (*n* = 3); ΔTER: 121.7 ± 27.6 Ω ⋅ cm^2^ (*n* = 3)].

**Fig. 5:**
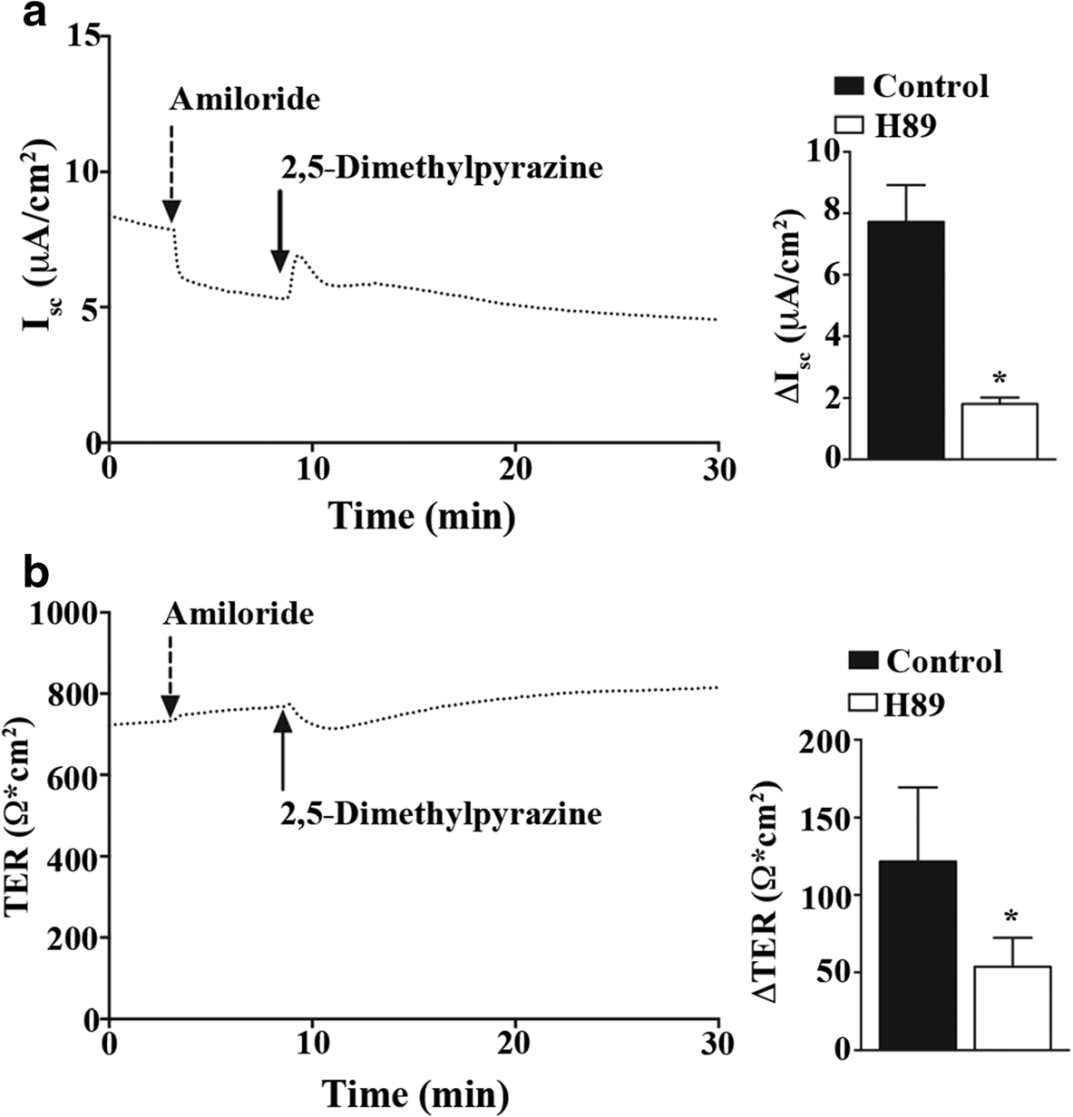
PKA signaling is upstream of 2,5-dimethylpyrazine-evoked apical ion efflux. Primary MTE cells were voltage clamped in Ussing chambers and monitored for changes in I_sc_ and TER following addition of 0.2 % v/v 2,5-dimethylpyrazine. **a** Representative traces of I_sc_ and **b** TER (left) from MTE cells pre-treated with H-89 followed by amiloride (dashed arrow) and 2,5-dimethylpyrazine (solid arrow). Quantification of I_sc_ and TER (A-B *righ*t) of amiloride controls (*n* = 3) versus cells treated with amiloride and H89 (*n* = 3) are graphed. Significant block of apical ion efflux with H89 suggests 2,5-dimethypyrazine-evoked apical ion efflux is mediated via PKA signaling. *indicates significant difference *P* < 0.05 in a one-way ANOVA with a Dunnett’s Multiple Comparison Test

In order to confirm that 2,5-dimethylpyrazine was acting through a cAMP-protein kinase A (PKA) signaling cascade, and not by directly activating CFTR, we used the PKA blocker H89. MTE cells were monitored for response to 2,5-dimethylpyrazine (0.2 % v/v; Fig. 5a-b solid arrow) following a 30 min pre-treatment with H89 (10 μM) and amiloride (10 μM; Fig. 5a-b dashed arrow). Representative I_sc_ and TER traces (dotted lines) for the blocking experiment are shown in Fig. 5a-b. The peak change in I_sc_ and TER evoked by 2,5-dimethylpyrazine in MTE cells pretreated with H89 [(Fig. 5a-b; ΔI_sc_: 1.8 ± 0.12 μA/cm^2^ (*n* = 3); ΔTER: 53.7 ± 10.8 Ω ⋅ cm^2^ (*n* = 3)] was significantly reduced compared to controls.

**Fig. 6:**
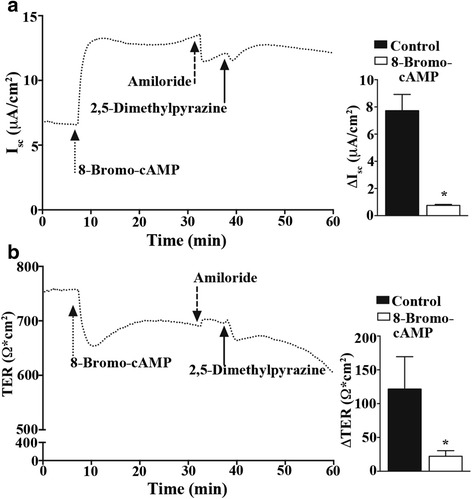
Increased intracellular cAMP dampens 2,5-dimethylpyrazine-evoked apical ion efflux. Primary MTE cells were voltage clamped in Ussing chambers and monitored for changes in I_sc_ and TER following addition of 0.2 % v/v 2,5-dimethylpyrazine. **a** Representative traces of I_sc_ and **b** TER *(left*) from MTE cells pre-treated with 8-bromo-cAMP (dotted arrow) followed by amiloride (dashed arrow) and 2,5-dimethylpyrazine (solid arrow). Quantification of I_sc_ and TER (A-B right) of amiloride controls (*n* = 3) versus cells treated with 8-bromo-cAMP and amiloride (*n* = 3) are plotted. The addition of the cAMP analog 8-bromo-cAMP significantly reduced apical ion efflux evoked by 2,5-dimethypyrazine suggesting it activates a receptor that increases cAMP production. *indicates significant difference *P* < 0.05 in a one-way ANOVA with a Dunnett’s Multiple Comparison Test

To test if an increase in intracellular cAMP concentration was necessary for the 2,5-dimethylpyrazine activation of CFTR in MTE cells, we administered the cAMP analog 8-bromo-cAMP (100 μM; Fig. 6a-b dotted arrow) to MTE cell cultures under current clamp conditions prior to the addition of 2,5-dimethylpyrazine. Following 8-bromo-cAMP addition, and upon I_sc_ and TER stabilization, we added amiloride (10 μM; Fig. 6a-b dashed arrow) and then 2,5-dimethypyrazine (0.2 % v/v; Fig. 6a-b solid arrow). Representative I_sc_ and TER traces (dotted lines) for this experiment are shown in Fig. 6a-b. The peak change in I_sc_ and TER evoked by 2,5-dimethylpyrazine in MTE cells pretreated with 8-bromo-cAMP and amiloride (Fig. 6a-b; ΔI_sc_: 0.8 ± 0.05 μA/cm^2^ (*n* = 3); ΔTER: 22.2 ± 4.8 Ω ⋅ cm^2^ (*n* = 3) was significantly reduced compared to controls. The addition of intracellular 8-bromo-cAMP significantly attenuated the changes in I_sc_ and TER evoked by 2,5-dimethylpyrazine, supporting the hypothesis that 2,5-dimethylpyrazine leads to increased levels of cellular cAMP to alter I_sc_. From these experiments we conclude that 2,5-dimethylpyrazine increases apical Cl^-^ efflux in primary airway epithelial cells via CFTR, and that activation of CFTR is through a cAMP-PKA signaling cascade.

## Discussion

In this study we used a high-capacity validated screen to examine concentration-dependent cytotoxicity from seven common e-cigarette flavorings in order to assess their safety in the airway. In addition to identifying cytotoxicity thresholds for five of the seven chemicals tested, we were able to identify two compounds, 2,5-dimethylpyrazine and vanillin, that significantly altered airway epithelial cellular physiologic responses. Further characterization of 2,5-dimethylpyrazine demonstrated a compromising effect on the response to common airway epithelial cellular signaling molecules in 16HBE14o- cells following a 24 h exposure. In primary MTE cells it evoked a rapid activation (min) of Cl^-^ current through a cAMP/PKA/CFTR-signaling pathway.

Early reports on e-cigarette toxicity focused primarily on identifying constituents in e-cigarette liquids and aerosols that are known to be harmful in conventional cigarettes (e.g., nicotine, tobacco-specific nitrosamines, aldehydes, volatile organic compounds) with less attention given to unique additives such as vegetable glycerin, propylene glycol and flavorings (for reviews see [3, 34, 35]). Limited studies on the pulmonary effects of short-term e-cigarette use include increased airway resistance, decreased exhaled nitric oxide, and reported symptoms of cough [36–38]. An important argument from these initial studies is the emphasis for controlled research to properly evaluate all components in e-cigarette aerosols and their impact on lung cells and tissues. Careful studies that outline toxicity of flavorings are especially important considering that taste, including the large varieties of flavors, is a key consideration that contributes to the use of e-cigarettes [36]. In this study we employed a high-capacity real-time cellular screening assay to identify those flavoring additives that demonstrate the potential to cause harm to the conducting airway epithelium. This high-capacity acute-liquid exposure model offers a means to delineate those flavoring constituents that are most detrimental from the multitude of constituents on the market. Such information can be used in future experiments that assess chronic aerosol exposures *in vitro* and *in vivo* to assess long-term effects.

Additives that allow for e-cigarette taste have been discussed as potential health hazards [13]. For example, an examination of flavoring constituents in 28 different e-liquid products found the presence of 141 different flavoring chemicals, some of which are known as allergenic compounds (e.g., eugenol and cinnamic aldehyde) [9]. An argument for the current use of flavorings in e-liquids is their prior approval by regulatory agencies for ingestion in small amounts. However, most chemicals used in flavorings have not been tested for respiratory toxicity via the inhalation route [39] and implications that ingestion safety is comparable to inhalation safety is, at best, misleading [40]. As an example, in the early 2000s several workers at microwave popcorn packaging plants across the U.S. developed bronchiolitis obliterans, a rare and irreversible obstructive lung disease that was later attributed to the artificial butter flavoring component diacetyl [12]. Despite the known inhalation toxicity of diacetyl, an examination of over 150 sweet flavored e-liquids found that 69.2 % contained diacetyl in both the e-liquid and its corresponding aerosol. Further, almost half (47.3 %) of these e-liquids contained diacetyl at concentrations above the National Institute for Occupational Safety and Health (NIOSH) safety levels for occupational exposure [41]. It is clear that a need for research to characterize both the presence of toxic chemicals in e-cigarette flavorings and the potential adverse respiratory effects of exposure to those flavorings is needed [13]. The experimental setup in this study aims to identify those flavoring chemicals that disrupt airway epithelial function and the mechanisms by which this disruption occurs.

It is becoming increasingly evident that constituents in e-liquids can compromise various aspects of airway epithelial innate immunity. In the absence of nicotine, e-liquids caused increased pro-inflammatory cytokines (e.g., IL-6) and increased human rhinovirus infection in primary human airway epithelial cells [42]. In a separate study, e-liquids containing flavorings, especially those with fruit or sweet flavors, were more oxidative than those without flavorings, and thus potentially more damaging to the airway [43]. These authors also found that e-liquid aerosols increased secretion of IL-6 and IL-8 from human airway epithelial cells grown at an air/liquid interface. Our studies using high-capacity real-time cell analysis show the e-liquid chemical 2,5-dimethylpyrazine reduces the ability of 16HBE14o- cells to respond to forskolin-induced cAMP signaling and to a lesser extent exogenous ATP at subcytotoxic concentrations. These signaling pathways underlie several important physiological functions in the conducting airway epithelium.

Our biophysical studies are indicative of an acute 2,5-dimethylpyrazine-evoked cAMP/PKA-signaling pathway that is consistent with the activation of an odorant receptor. Odorant receptors are not restricted to the upper airway and can have distinct physiologic function within the conducting airway as well as in other parts of the body (reviewed in [44]). 2,5-dimethylpyrazine is also a potent pheromone in both *Drosophila* [45] and in mice where it can lead to suppression of reproductive activities (reviewed in [46]). In this study we show that 2,5-dimethylpyrazine activates cAMP/PKA signaling leading to transient changes in short-circuit current and transepithelial resistance via CFTR in mouse conducting airway epithelial cells. Chronic activation of CFTR signaling *in vivo* may alter CFTR expression and salt and water balance in the airway lumen that could negatively impact airway epithelial cell innate immune mechanisms such as mucociliary clearance.

## Conclusions

E-cigarettes have become a common way to introduce flavorings via inhalation with unknown long-term health effects. It is established that flavored conventional cigarettes are more appealing among youth; 17 year olds three times more likely than 25 year olds to smoke a flavored cigarette [47]. Because of such findings, the 2009 Family Smoking Prevention and Tobacco Control Act banned flavored conventional cigarettes (excepting menthol and tobacco flavor) in an effort to reduce the number of young adults who become addicted to cigarettes [48]. The recent introduction of ENDS, including e-cigarettes has provided an avenue to re-introduce flavorings to inhalation devices. With the multitude of e-cigarette flavoring choices in the marketplace it is essential that the constituents comprising these flavorings be assessed for human safety in order to inform regulatory authorities, healthcare providers and most importantly, e-cigarette users. Our approach to screen constituents for both cytotoxicity and subcytotoxic alterations in conducting airway epithelial cell physiology, followed by mechanistic studies, provides a successful strategy for understanding potential toxicants commonly used in e-cigarettes and other ENDS.
